# Supplementary material for: Psychological well-being of SLE patients: A comprehensive examination of stress, depression, anxiety and quality of life
Source: PLoS One. 2026 May 11;21(5):e0344330. doi: 10.1371/journal.pone.0344330 (PMC13160299; doi:10.1371/journal.pone.0344330)
Supplement: Attachment — Submitted filename: Response to Reviewers_Rebuttle V2.doc [file pone.0344330.s003.doc]

**Response to Reviewers**

On behalf of my co-authors, I would like to sincerely thank the reviewer(s) for their constructive and valuable comments on the various sections of our manuscript. It truly helped in improving the quality of the manuscript. We greatly appreciate reviewer’s time and effort in reviewing our work.

In response to the reviewer’s suggestions, we have prepared this document, as a ‘response to reviewers’, and organized it pointwise. The changes have been incorporated into the main manuscript and are highlighted using track changes for ease of reference. I have also pasted excerpts from the manuscript where required and possible.

Below is a pointwise summary of our responses to the reviewer’s comments:

**Reviewer 2:**

**Comment 1: Point 1:** Results indicate the prevalence of stress and generalized and trait anxiety among the SLE patients, except for depression. (Please mention whether prevalence is high or low)

**Response**: Thank you for highlighting this. The text has been revised. The revised content is presented below in italics:

*“Results indicate a high prevalence of stress, depression, and both generalized and trait anxiety among the SLE patients”.*

**Point 2:** Page 2,Line 22 - When compared with rheumatoid arthritis (another autoimmune disease) and without autoimmune conditions, SLE patients reported higher distress than others.

Response: The text has been revised. Please refer to the lines, 22 -23 in the manuscript without track-changes.

**Comment 2: In the introduction, the authors describe the prevalence of SLE as 3.2 - 3000/100,000 individuals. The reference quoted does not give such a high prevalence. Please correct it.**

**Response:** The prevalence figures have been revised. We’ve also added a recent reference to highlight the wide range of reported incidence and prevalence. Please refer to the lines 36 -40 in the revised manuscript.

**Comment 3:** Second paragraph of the Methods section on Page 2 describing participant details should be part of the Results section

**Response:** This has been revised. Please refer to the lines, 249-259.

**Comment 4:** The statement from the consent form can be included as part of the annexure and does not need to be a part of the main manuscript (Procedure)

**Response:** We have chosen to retain this section in the manuscript to highlight the best practices in participant consent.

**Comment 5:** The cluster differences highlighted in the discussion are not sufficiently wide between the two groups to yield any clinical utility. The age difference between the two groups is only 3 years (25 in Cluster 1 and 28 in Cluster 2). Technically, both clusters are young. Similarly, with regard to occupation, unemployment rates were abnormally high in both clusters (73% in the lower cluster). Please mention the unemployment rates in both clusters. Also, the difference may be statistically significant, but it does not appear to be clinically meaningful. Please discuss this in the limitations in the discussion on cluster analysis. This could be due to a small sample size.

**Response:** Thank you once again for these valuable suggestions. The discussion section has been revised. Please refer to the revised sections from line, 397-420 in the revised manuscript. Below the revised text in italics:

*Cluster 2 (n = 48) comprised comparatively older participants (median age = 29 vs. 23 years) with a higher proportion of homemakers, resulting in fewer individuals with formal employment. Specifically, 73% of participants in Cluster 2 were homemakers, 17% were formally employed, and 10% were students, whereas Cluster 1 (n = 54) included 39% homemakers, 15% were formally employed participants, and 46% were students. The older age profile and higher proportion of participants without formal employment in Cluster 2 may help explain the poorer psychological health scores observed in this group compared with the younger, student dominated Cluster 1. These observations resonate with prior studies emphasizing the impact of unemployment on mental health, particularly in regions with limited economic development [42]. Additionally, participants in Cluster 2 reported lower quality of life across physical, psychological, social, and environmental domains, consistent with previous research linking quality of life to the mental health of SLE patients [43].*

*While these differences may not be directly clinically relevant, they provide insight into the demographic factors contributing to the quality-of-life experiences associated with SLE. These findings are consistent with previous reports highlighting demographic disparities in SLE burden, demonstrating higher incidence and prevalence rates of SLE among Black, Hispanic, and Asian populations compared with White Americans [13]. Overall, these findings highlight the heterogeneous nature of psychosocial well-being among SLE patients and emphasize the importance of considering demographic factors in future research aimed at elucidating the multifaceted contributors to this complexity.*

*Despite the promising results, a larger sample size would be required to enhance the robustness of the clusters, with multiple clustering methods used to better capture the underlying structure of complex psychological traits.*

**Reviewer 2:** If possible please address the relation between neuropsychiatric SLE ,your results and any available related markers ,also as regards anxiety family members and caregiver inclusion in the study will add much to the objectivity of the results.

**Response:** Thank you for this suggestion. The current study included only the PHQ, GAD, STAI-T, WHO Brief, and BILAG assessments. However, your recommendations regarding additional markers, and familial history are highly valuable, and we will consider them in our future research and analyses.
